# Aurora kinase-a expression heterogeneity and potential benefit of combination therapy in prostate adenocarcinoma

**DOI:** 10.3389/fcell.2025.1608711

**Published:** 2025-07-11

**Authors:** Ru Chen, Qianyi Qiu, Weiting Xie, Jun Lin, Rong Liu, Jianhui Chen, Shaoxing Zhu, Yiming Su

**Affiliations:** Department of Urology, Fujian Medical University Union Hospital, Fuzhou, China

**Keywords:** AURKA, heterogeneity, prostate adenocarcinoma (PRAD), AURKA inhibitor, combination therapy

## Abstract

**Background:**

Aurora kinase A (AURKA) is aberrantly expressed in a large number of tumors and promotes tumor progression by regulating the cell cycle, chromosomal instability, and drug resistance. However, its heterogeneous expression and combination therapy benefit in prostate adenocarcinoma (PRAD) is unclear.

**Methods:**

In this study, we integrated TCGA pan-cancer multi-omics data and GEO data to analyze the RNA, methylation, protein expression, and genomic alteration characteristics of AURKA. We then used single-cell RNA sequencing to resolve the functional heterogeneity of AURKA in the PRAD epithelial cell subpopulation and verified its impact on the malignant phenotype of desmoplasia-resistant prostate cancer cells in in vitro experiments. This research also analyzed the prognostic risk stratification of AURKA subpopulations in combination with various indicators and the potential benefit of AURKA inhibitors in combination with various treatments.

**Results:**

The pan-cancer analysis demonstrated that AURKA expression heterogeneity was present among urological tumors at different molecular levels, and the positive correlation of AURKA alteration with MYC and E2F pathways was conserved in pan-cancer. Epithelial cell subpopulations with high expression of AURKA (epi3/4/6) promoted proliferation by regulating cell cycle and DNA repair, while low expression subsets (epi1/2/7) activated TNF-α and androgen receptor (AR) pathways to mediate drug resistance. In particular, AURKA may serve as a compensatory pathway to support tumor activity after AR inhibition in prostate cancer, a complex mechanism not seen in other tumors. AURKA-overexpressing patients with low Gleason scores or high PSA have a poor prognosis in clinical analysis. Furthermore, a comprehensive drug sensitivity co-analysis found that AURKA inhibitors may benefit from targeted therapy, ADC therapy, and immunotherapy. TMB and CD274 expression were the biomarkers of AURKA high-expression patients with PRAD for clinical outcome.

**Conclusion:**

AURKA expression heterogeneity has been identified as a critical factor in the progression of PRAD and the development of drug resistance. The molecular subtyping of AURKA can serve as a precise strategy for combination therapy and provide a theory for the combination of AURKA inhibitors and targeted/immunotherapy.

## 1 Introduction

Aurora Kinase-A (AURKA) is a serine/threonine kinase that is highly expressed in various tumor types (Du et al., 2021). Previous studies have shown that AURKA promotes aberrant proliferation of tumor cells mainly by regulating centrosome maturation and segregation during mitosis, leading to chromosomal instability and cell cycle deregulation (Lin et al., 2020; Willems et al., 2018; Polverino et al., 2024; Cacioppo and Lindon, 2022). AURKA overexpression has been found to correlate with metastasis and poor prognosis in several cancers, suggesting that it is a promising target for treatment and a prognostic biomarker (Du et al., 2021; Yang et al., 2023). The functional role of AURKA has been extensively studied in breast (Fuentes-Antrás et al., 2024), lung (Chang et al., 2024), and gastrointestinal (Cheng et al., 2021; Zhou et al., 2024)cancers, but its understanding of prostate adenocarcinoma (PRAD) is still lacking.

Recent studies have demonstrated that AURKA can not only promote tumor progression but is also associated with drug resistance. AURKA phosphorylates and inhibits p53 activity, promoting apoptosis and cell survival (Naso et al., 2021). Furthermore, AURKA induces epithelial-mesenchymal transition (EMT) and promotes tumor cell migration and invasion by activating signaling pathways such as PI3K/AKT and Wnt/β-catenin (Grisetti et al., 2024; Lv et al., 2024). In terms of its role in promoting drug resistance, AURKA is associated with resistance to several chemotherapy drugs (e.g., paclitaxel, cisplatin) (Cheng et al., 2021; O'Shaughnessy et al., 2021; Li et al., 2025). AURKA-based resistance may involve increased DNA repair capacity, activation of autophagy, and upregulation of anti-apoptotic pathways (Zheng et al., 2023; Shao et al., 2023). In addition, AURKA may also enhance tumor escape by modulating the tumor immune microenvironment (Liu et al., 2025). However, further research is required to fully elucidate the clinical significance of AURKA abnormalities at different omics levels. Prostate cancer is a highly heterogeneous hormone-driven malignancy (Kneppers et al., 2022). The prognosis prediction of AURKA-driven molecular subtypes in different populations has considerable potential to guide the development of precision clinical therapy in the future.

AURKA small-molecule inhibitors (e.g., alisertib) have entered clinical trials, while many challenges remain. AURKA inhibitor-based clinical trials have been reported in several solid tumors with partial success (Haddad et al., 2018; Falchook et al., 2019; Zhang et al., 2024; Haddad et al., 2023). However, a study of AURKA inhibitors in prostate cancer suggests that monotherapy with AURKA inhibitors is not effective in improving patient prognosis which may be partly due to compensatory survival pathways (Beltran et al., 2019). These studies suggest that AURKA inhibitor-based therapy requires precise stratification in patients with prostate cancer, and combination therapy is a future trend in prostate cancer (Nikhil and Shah, 2024).

In this study, we revealed the heterogeneity of AURKA expression in epithelial cells and the potential benefits of combination therapy for prostate cancer. The results demonstrated that AURKA expression exhibited a positive correlation with tumor malignancy and high expression was associated with poor prognosis. The results indicated that the high-expressing epithelial cells were highly heterogeneous. The abnormally high AURKA-expressing subset promoted tumor cell proliferation and migration by regulating cell cycle and mitosis. In contrast, the relatively low AURKA-expressing subset regulated the TNF-α and the ANDROGEN (AR) response pathway to induce drug resistance. Notably, the high-expressing AURKA group showed a better prognosis in PSA-high and Gleason-low for prostate cancer patients. Furthermore, we have predicted that the AURKA high-expressing population may be suitable for combination with target, ADC, and immune therapy. The accurate population stratification of AURKA subgroups in prostate cancer can facilitate personalized treatment.

## 2 Methods

### 2.1 Data collection

The RNA, DNA, methylation and protein data of the The Cancer Genome Atlas Consortium (TCGA) pan-cancer expression data were all downloaded from Xenabrowser of GDC hub. Bulk RNA-seq cohorts were downloaded from Gene Expression Omnibus (GEO) websites (GSE46602 (Sun et al., 2021), GSE54460 (Long et al., 2014), GSE70768 (Ross-Adams et al., 2015) and GSE70769 (Ross-Adams et al., 2015) and Cbioportal (prad_mskcc (Taylor et al., 2010) and prostate_dkfz_2018 (Gerhauser et al., 2018)). Single-cell datasets from prostate cancer tumor samples used in this study include: GSE137829 (Dong et al., 2020), GSE141445 (Chen et al., 2021), GSE157703 (Ma et al., 2020), GSE181294 (Hirz et al., 2023), GSE185344 (Wong et al., 2022), GSE193337 (Heidegger et al., 2022), GSE206962 (Cheng et al., 2024), GSE221603 (Huang and Yin, 2025), GSE230282 (Watanabe et al., 2023), GSE250189 (Pan et al., 2024), GSE264573 (Zaidi et al., 2024).

### 2.2 Single cell data processing and batch correction

Single-cell RNA sequencing (scRNA-seq) data were processed using the Seurat package (version 4.1.0) in R (Satija et al., 2015), with each sample subjected to the following filtering criteria: genes expressed in at least three cells were retained (min.cells = 3), cells with ≥200 detected genes were included (min.features = 200), and cells exhibiting >15% mitochondrial gene content were excluded (percent.MT < 15%). To address batch effects, data were standardized via the scaleData function in Seurat, followed by identification of the top 2,000 variable genes using FindVariableFeatures. Batch correction was performed through Principal Component Analysis (PCA) combined with Harmony (version 1.2.3) (Korsunsky et al., 2019), after which Uniform Manifold Approximation and Projection (UMAP) visualization was applied based on Harmony-reduced dimensions. The number of principal components (PCs) was determined using the ElbowPlot function in Seurat, and cell sub-clustering was conducted using FindNeighbors and FindClusters algorithms. Cell clusters were annotated based on canonical marker genes, including PTPRC (CD45) for lymphocytes; CD4, CD8A, FCGR3A (CD16), and NCAM1 (CD56) for T/NK cells; CD19 and MZB1 for B/plasma cells; CD68 and TPSAB1 for myeloid/mast cells; EPCAM for epithelial cells; VWF for endothelial cells; and FAP for fibroblasts.

### 2.3 Identification of malignant epithelial cells

Cell differentiation initial states and evolutionary directions were defined using CytoTRACE (version 1.0.0) (https://github.com/digitalcytometry/cytotrace2/releases), used for the assessment of cancer cell stemness.

Malignant copy number variation (CNV) events in epithelial cells were determined via the inferCNV package (version 1.14.2) (GitHub, 2023). In this workflow, T cells were designated as the spike-in reference control population, with the minimum average read count per gene among reference cells set to 0.1. Clustering was performed using Ward.D2 hierarchical clustering, and CNV prediction was executed in denoising mode. Genes exhibiting fold change values below 0.9 or exceeding 1.1, as determined by the distribution of fold change metrics, were classified as CNV events.

### 2.4 Differential expression analysis and functional enrichment analysis

Differential gene expression analysis was implemented using the Seurat package’s FindMarkers function to delineate cluster-specific marker genes, with selection criteria encompassing three thresholds: (1) gene expression prevalence exceeding 25% within the focal cell cluster, (2) a log2 fold change (log2FC) ≥ 0.25, and (3) an adjusted p-value threshold of <0.05. For functional annotation, Kyoto Encyclopedia of Genes and Genomes (KEGG) pathway enrichment was executed via the clusterProfiler package (v4.12.1) (Yu et al., 2012), with the top five enriched pathways per cluster selected to define their biological roles. Pseudo-bulk expression profiling involved first computing mean transcript levels per sample using the AverageExpression function, followed by differential expression testing through limma (v3.56.2) (Smyth et al., 2005). Gene Set Enrichment Analysis (GSEA) for Gene Ontology (GO) terms was performed using clusterProfiler’s gseGO function, while pathway-based clustering patterns were visualized via the aPEAR package (v1.0.0) (Kersev et al., 2023).

### 2.5 Functional gene set expression analysis

To evaluate functional gene set expression in scRNA-seq data, the irGSEA package (v2.1.5) (Fan et al., 2024) was employed to analyze three predefined gene set categories: (1) the built-in tumor hallmark gene set, (2) a curated myeloid cell functional status gene set, and (3) a fibroblast cell functional gene set. For pathway expression quantification, both UCell and AUCell methods were adopted (Andreatta and Carmona, 2021), with results from these complementary approaches integrated to ensure reliability. Only pathways demonstrating concordant significance (Wilcoxon test, two-tailed *p*-value < 0.05) across both methods were subsequently depicted in a cluster-specific hallmark pathway expression heatmap.

### 2.6 Transcription factor (TF) analysis

Transcription factor (TF) regulatory activity was analyzed using the SCENIC (v1.3.1) and pySCENIC (v0.11.2) pipelines (Aibar et al., 2017). The workflow proceeded in three stages: (1) GRNBoost2 was first applied to construct a gene regulatory network, identifying potential target genes regulated by each TF; (2) RcisTarget was then utilized to enrich transcription factor binding motifs within the regulatory regions of these targets; and (3) regulon activity quantification was performed via AUCell, with thresholds for significant TF activation determined automatically based on area under the curve (AUC) metrics (activation in ≥20% of cells).

For regulon-based clustering, the Connection Specificity Index (CSI) was employed to identify five distinct co-expression modules. Subsequently, the calcRSS algorithm was applied to pinpoint TFs specifically associated with subcluster identities and drug resistance phenotypes. To elucidate functional implications, KEGG pathway enrichment analysis was conducted on PPARX2 and its high-confidence target genes (co-expression network edge weights >10), revealing candidate regulatory pathways involved in these biological processes.

### 2.7 Drug sensitivity analysis

Drug responsiveness was evaluated using the GDSCv2 database (https://www.cancerrxgene.org/), with inter-group IC50 discrepancies analyzed via the oncopredict package (Geeleher et al., 2017). For pathway-level drug-target enrichment analysis, hypergeometric statistical testing was implemented, incorporating Benjamini–Hochberg adjustment to control the false discovery rate (FDR) of p-values.

### 2.8 Cell cultures and transfection

Human prostate cancer cell lines (PC3, DU145, and LnCAP) were purchased from the Cell Resource Center Affiliated with the Chinese Academy of Medical Sciences. These cell lines were resuscitated, subsequently subjected to centrifugation, and resuspended in complete medium (DMEM supplemented with 10% fetal bovine serum (FBS) and 1% penicillin-streptomycin). The culture medium was changed daily based on cell density, and cells were passaged every 3 days. When cells reached the exponential growth phase, they were passaged at a ratio of 1:3. For gene modulation, small interfering RNAs (siRNAs) targeting AURKA and non-targeting negative control siRNA (siCtrl) were purchased from GenePharma (Shanghai, China). The siRNA sequences used were as follows: AURKA-siRNA (1#,5′-CCTGTCTTACTGTCATTCGAA-3’; 2#,5′-GAGTCTACCTAATTCTGGAAT-3’; 3#,5′-CACATACCAAGAGACCTACAA-3′),

siCtrl: 5′-TTCTCCGAACGTGTCACGT-3′. The siRNAs were added to cells using Lipofectamine™ 3000 reagent for gene silencing experiments. The cells were then cultivated in a cell incubator to conduct downstream experiments.

### 2.9 Clone formation assay

Treated cells were incubated onto six-well plates (1000 cells per well) cultured for 2 weeks. Subsequently, colonies were fixed with paraformaldehyde and stained with crystal violet. Finally, images were captured using a digital camera, and clones were counted. This experiment was repeated at least three times.

### 2.10 Migration and invasion assays

Cell migration and invasion were evaluated using the Transwell ® system (Corning, Inc., Corning, NY). For assay preparation, the lower chamber was filled with 500 μL complete medium (Ham’s F-12K and MEM medium supplemented with 10% fetal bovine serum (FBS) and 1% penicillin/streptomycin (P/S)). Following a 24-h incubation period, cells were washed with phosphate-buffered saline (DPBS, Servare Biotech Inc., Hangzhou, Zhejiang, China), fixed with absolute ethanol, and stained with 0.1% crystal violet (Sigma-Aldrich, St. Louis, MO) for 20 min. Finally, migrated/invasive PC3 and DU145 cells were quantified under an inverted microscope (ICX41, Ningbo Sunny Instruments Co., Ltd., Yuyao, Zhejiang, China).

### 2.11 Statistical analysis

Statistical analyses were performed using R version 4.3.1. Survival outcomes were evaluated using the Survival (v3.8.3) (Therneau and Grambsch, 2000) and Survminer (v0.5.0) (Kassambara et al., 2024) packages for survival analysis and visualization. Kaplan-Meier survival curves were generated to compare survival outcomes, while Cox proportional hazards regression models were fitted to evaluate survival associations. The cox-ph model was applied for survival testing, and forest plots were constructed using the forestplot package (v3.1.6) to visualize hazard ratios and confidence intervals.

Differential gene expression between control and knockdown groups was assessed using Student’s t-test, with paired comparisons analyzed via the Wilcoxon signed-rank test. Spearman’s rank correlation coefficient was utilized to quantify nonlinear associations between variables. Group comparisons involving two cohorts were performed using the Wilcoxon rank-sum test, and all correlation analyses employed Spearman’s nonparametric method. Results are presented as mean ± standard error of the mean (SEM), with statistical significance defined as *p* < 0.05.

## 3 Results

### 3.1 AURKA expression inter-tumor heterogeneity across pan-cancer

**FIGURE 1:**
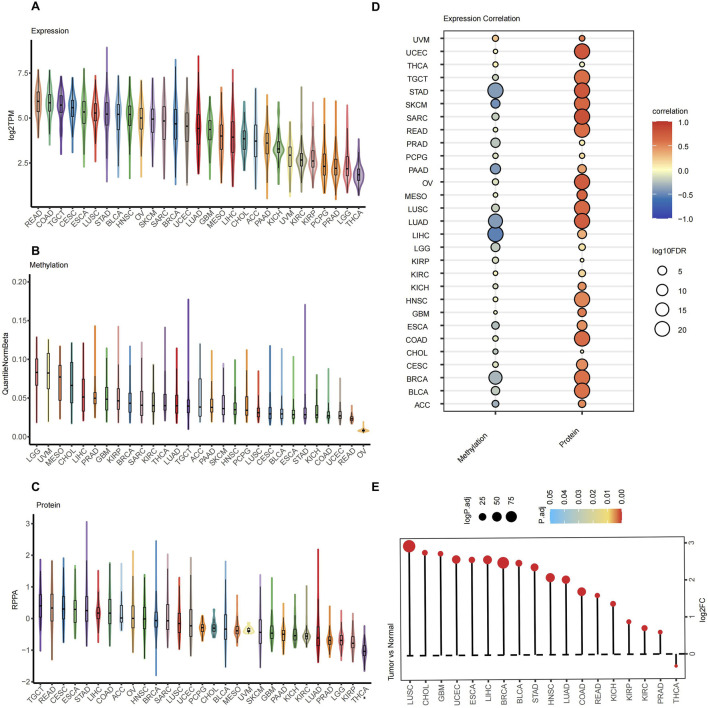
AURKA expression at various molecular levels in pan-cancer. Differential **(A)** mRNA, **(B)** methylation and **(C)** protein expression of AURKA in pan-cancer. **(D)** The Spearman correlation between AURKA mRNA expression and DNA methylation levels (left column), as well as between AURKA mRNA and protein expression levels (right column) in pan-cancer. **(E)** Differential mRNA expression of AURKA in cancer compared with normal tissues.

A comprehensive analysis of AURKA expression was performed in 33 cancer types, comparing normal and tumor samples from the TCGA dataset. The research revealed significant variations in the different molecular levels of AURKA expression across pan-cancer, such as at the RNA, methylation, and protein levels. AURKA expression was higher in gastrointestinal tumors (e.g., READ and COAD) and lower in endocrine or nervous system tumors (e.g., THCA and LGG) at the RNA level (Figure 1A). The AURKA expression was highly heterogeneous across urinary tract tumors, with higher expression in TGCT and lower in PRAD. Conversely, neuroendocrine tumors (LGG) showed higher levels of methylation (Figure 1B). Urological tumors exhibited comparatively lower methylation levels, while PRAD expression was high. A negative correlation between methylation and RNA expression was observed in most cancers, which was consistent with the prevailing conventional understanding. Among the examined tumors, the negative correlation was most pronounced in gastrointestinal tumors (e.g., STAD, PAAD), PRAD, LUAD, and BRCA (Figure 1D). At the protein expression level, gastrointestinal cancer (e.g., READ, ROAD) showed relatively higher expression, while the urinary system (e.g., PRAD) and LUAD tumors showed lower expression (Figure 1C). In the majority of tumors, AURKA RNA levels showed a positive correlation with protein expression. However, the correlation was weak in THCA, PRAD, KIRP, and CHOL (Figure 1D). Nevertheless, AURKA expression was found to be upregulated in the majority of tumors (Figure 1E), suggesting a critical role in tumor development and progression.

**FIGURE 2:**
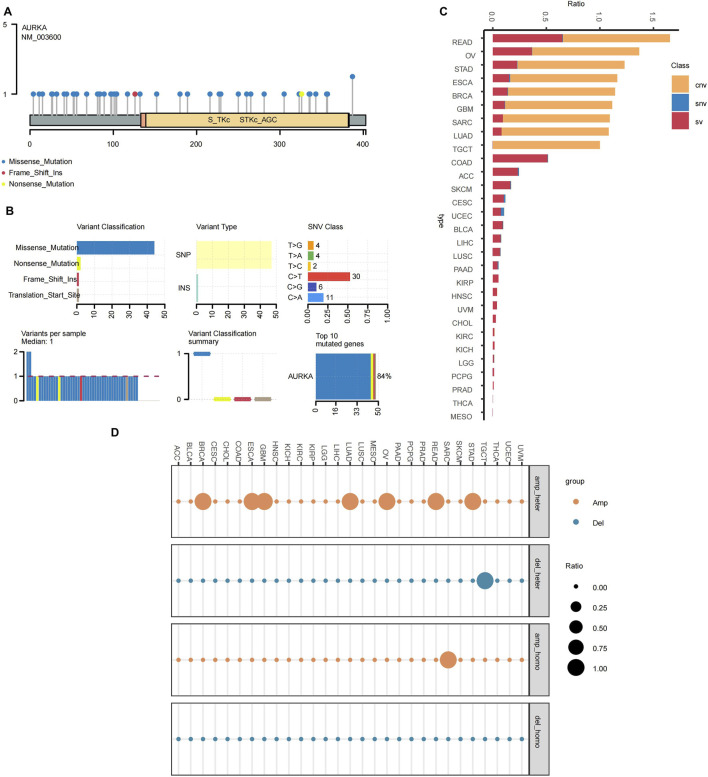
Analysis of the AURKA variations in pan-cancer. **(A)** AURKA variation distribution and **(B)** variation analysis. **(C)** Structural variation of AURKA in pan-cancer. **(D)** Amplification and deletion of AURKA in pan-cancer.

Next, we investigated the genomic characteristics of AURKA in pan-cancer. The distribution of gene mutation sites indicated that AURKA mutation sites were dispersed along the entire gene sequence, with certain regions exhibiting a higher mutation density (Figure 2A). Further analysis of the AURKA mutation preferences reveals that missense mutations are the predominant mutation type, accounting for a much higher proportion than other types such as nonsense mutations, insertions/deletions, etc. Among the various mutation types, single-nucleotide variants (SNVs) and the C>T transition were the most prevalent (Figure 2B). As illustrated in Figure 2C, a clear pattern of AURKA alterations was observed in different cancer types. The predominant variation types of AURKA were CNVs (amplification/deletion) and Structural Variants (SVs), with the proportion of SNVs being the lowest. Notably, high-frequency CNVs were observed in BRCA and TGCT, while SVs were significantly enriched in COAD and ACC. AURKA exhibited both CNV amplification and SV abnormalities in READ and OV, suggesting that its genomic instability may be related to specific tumor microenvironments. Furthermore, our findings revealed that READ, OV, and BRCA showed mainly heterozygous amplifications, whereas the frequency of heterozygous deletions was significantly higher in TGCT than in other cancers when analyzing the CNV situation of AURKA (Figure 2D). Taken together, AURKA showed no significant genetic variation in urological cancer (e.g., PRAD) (Figures 2C,D).

The profile of AURKA was heterogeneous, and its function affects or depends on the specific tumor microenvironment (TME). The heterogeneity is particularly evident in urologic tumors, where the mechanism of AURKA involvement may be more complicated.

### 3.2 Association between AURKA and immune infiltration in pan-cancer

**FIGURE 3:**
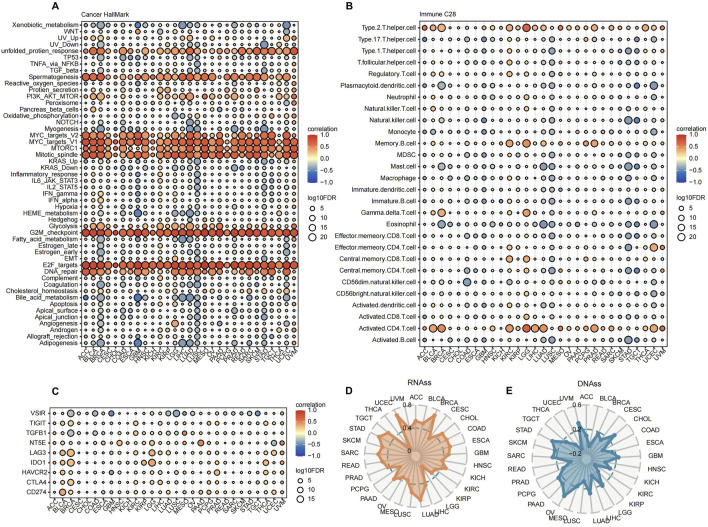
The factors associated with AURKA and the immune microenvironment. **(A)** Bubble plot of AURKA’s association with Hallmark pathways and **(B)** Bubble plot of AURKA’s association with 28 types of immune cells, as well as **(C)** immune checkpoints in pan-cancer. **(D)** Relationship between AURKA expression and DNA Stemness Score (DNAss) and **(E)** RNA Stemness Score (RNAss) in pan-cancer.

We investigated the correlation of AURKA with the tumor immune microenvironment (TIME) to uncover insight into the biological pathways involved in AURKA. In the correlation analysis with hallmark pathways, the AURKA expression had a strong and positive correlation with pathways such as MYC targets, G2M checkpoint, and E2F targets in the majority of cancers (correlation coefficients > 0.6) (Figure 3A). This observation suggested a degree of conservatism in the specific function of AURKA. Furthermore, the expression levels of AURKA in different cancers showed discrepant correlations with 28 immune cell types. Notably, AURKA expression showed a predominantly positive correlation with activated CD4^+^ T cells and T helper 2 cells across cancer types (Figure 3B). In addition, AURKA expression was significantly and positively correlated with immune checkpoint targets such as CD274, LAG3, and CTLA4 in most cancer types (Figure 3C). The results revealed that AURKA was actively involved in the tumor immune microenvironment (TIME). The observed positive correlation between activated CD4^+^ T cell levels and the expression of most immune checkpoint inhibitor targets provides a theoretical basis for combining AURKA inhibitors with other treatments.

Tumor stem cells are characterized by their ability to proliferate indefinitely, metastasize, and resist chemical toxicants. The stemness index serves as an indicator of the similarity between tumor cells and stem cells, which is associated with a higher degree of tumor dedifferentiation (Zhang et al., 2020; Mengistu et al., 2024). A correlation analysis between AURKA and cancer stemness at the RNA level revealed a coefficient of 0.7 for UCEC and BRCA (Figure 3D). At the DNA level, for OV and UVM, the correlation coefficients were 0.35 and 0.28, respectively (Figure 3E). This finding suggested that AURKA overexpression promoted tumor progression. Our analysis confirmed that AURKA-high had a worse prognosis (Supplementary Figure 1).

Hence, further analysis of the mechanisms and roles of AURKA in different subsets may help us in precision therapy with AURKA inhibitors, especially in urological tumors such as prostate cancer.

### 3.3 AURKA expression heterogeneity in epithelial cells through single-cell RNA analysis in PRAD

**FIGURE 4:**
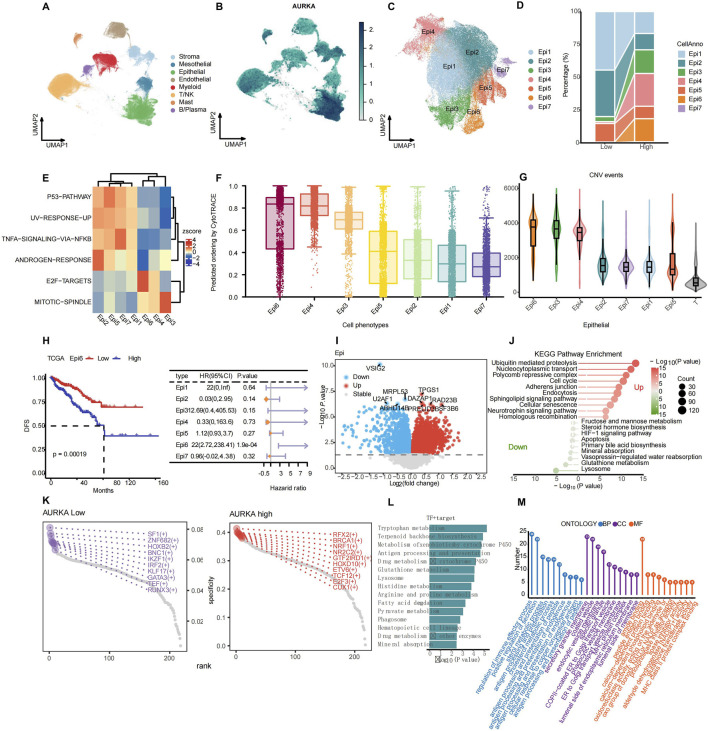
Characterization analysis of AURKA expression and cancer stemness-related subpopulations in scRNA-seq data. **(A)** UMAP plot of total cell annotation of merged PRAD single-cell data, different colors represent different cell types and **(B)** the distribution of AURKA expression. **(C)** UMAP plot of the epithelial cell (Epi) subpopulations of merged PRAD single-cell data. **(D)** Proportion of each Epi subpopulation in low- and high-AURKA expression groups. **(E)** Heatmap of hallmark pathway enrichment scores (UCell) across epithelial subpopulations. **(F)** Histogram chart comparing the cancer stemness of epithelial cell subtypes evaluated by CytoTRACE. **(G)** Violin plot of malign CNV events across epithelial cell subpopulations. **(H)** Association between Epi 6 expression levels and disease-free survival (DFS), as well as the forest plot of DFS prognostic analysis of Epi subsets. **(I)** DEGs of high CSCs Epi (Epi 3/4/6) group and low CSCs Epi (Epi 1/2/5) group. **(J)** Lollipop plot of differentially enriched KEGG pathways between high and low CSCs Epi. **(K)** Rank points of transcription factors (TFs) specifically enriched in AURKA low and AURKA high groups. **(L)** KEGG pathway enrichment of TFs and their target genes. **(M)** GO pathway analysis of TFs and their target genes: biological process (BP, blue), cellular component (CC, purple), and molecular function (MF, orange).

We explored the heterogeneous expression of AURKA and its mechanism and function in PRAD. The analysis of single-cell RNA results revealed that AURKA was distributed widely among different cell types, with the highest expression observed in epithelial cells (Figures 4A,B; Supplementary Figure 2A). UMAP map showed that AURKA expression levels were significantly increased in Epi3, Epi4, and Epi6 subsets (Figure 4C; Supplementary Figure 3). Based on the mean value of AURKA expression in each sample compared with the mean value of AURKA expression in the overall epithelial cells, we divided the samples into the AURKA high and low expression groups. Further analysis indicated that the proportion of Epi3, Epi4, and Epi6 subsets was higher in the AURKA-high group (Figure 4D). Our results showed that Epi6, Epi4, and Epi3 were enriched mainly in E2F and MITOTIC pathways, which are mainly associated with cell cycle regulation, mitosis, and DNA repair (Figure 4E). These subsets had higher cell stemness and CNV events, suggesting that these AURKA-expressing subsets may promote tumor development and progression (Figures 4F,G). However, Epi1, Epi2, and Ep7 were mostly enriched in the TNF-α and ANDROGEN response pathways, suggesting that they were associated with drug resistance (Figure 4E). Moreover, the results showed that AURKA may be involved in the compensatory mechanism of maintaining tumor aggressiveness after ANDROGEN deprivation therapy. In addition, deconvolution analysis revealed that different AURKA-driven epithelial cell subsets had different clinical prognoses. Among them, Epi6-high had a worse prognosis, and the percentage of Epi6 could be used as a prognostic marker for prostate cancer (Figure 4H).

The epithelial cells were divided into high and low Cancer Stem Cell (CSCs)-epi groups according to the median stemness score. By combining Epi3, Epi4, and Epi6 into one group, we found that there were different genes between them and the remaining subtypes (Figure 4I), and these genes are mainly involved in biological processes such as cell cycle regulation, DNA repair, and cell adhesion. KEGG pathway enrichment analysis further elucidated the critical role of these genes in activating key pathways, including “cell cycle,” “mismatch repair,” and “p53 signaling pathway” (Figure 4J). Additionally, the analysis identified specific co-expressed transcription factors (TFs), including RFX2 and BRCA1, that play critical roles in cell cycle regulation and differentiation (Figure 4K). Further analysis of the KEGG pathway enrichment of these TFs and their target genes reveals that they are involved in the regulation of pathways such as “cell cycle,” “apoptosis,” and “drug metabolism” (Figure 4L). Furthermore, Gene Ontology (GO) pathway analysis indicates that high expression of AURKA is associated with the regulation of antigen presentation-related pathways (Figure 4M). PPI network analysis revealed that AURKA was located in a relatively central position in the network and was closely associated with ANXA family genes, which are involved in antigen presentation, immune recognition (such as HLA-E, HLA-A, B2M), and signal transduction (such as HSP90AA1, TMP1, VIM). These interactions suggested the potential for AURKA to regulate cell adhesion, signal transduction, and immune response through its influence on these genes. In addition, the presence of cytoskeletal proteins (such as VIM) and signaling molecules (such as EFHD2) in the network suggested that AURKA may affect cell migration and invasion by regulating cytoskeletal remodeling and signaling (Supplementary Figure 2B).

The expression of AURKA in epithelial cells exhibited significant heterogeneity. Its high expression in specific epithelial cell subgroups is closely related to multiple malignant phenotypes of tumor cells, such as proliferation capacity and stemness characteristics. The potential interaction of AURKA with the AR pathway provided the complexity of tumor progression and drug resistance. Therefore, in-depth research on AURKA will help to analyze its function and mechanism in different populations.

### 3.4 Validation of AURKA for promoting tumor progression in prostate cancer

**FIGURE 5:**
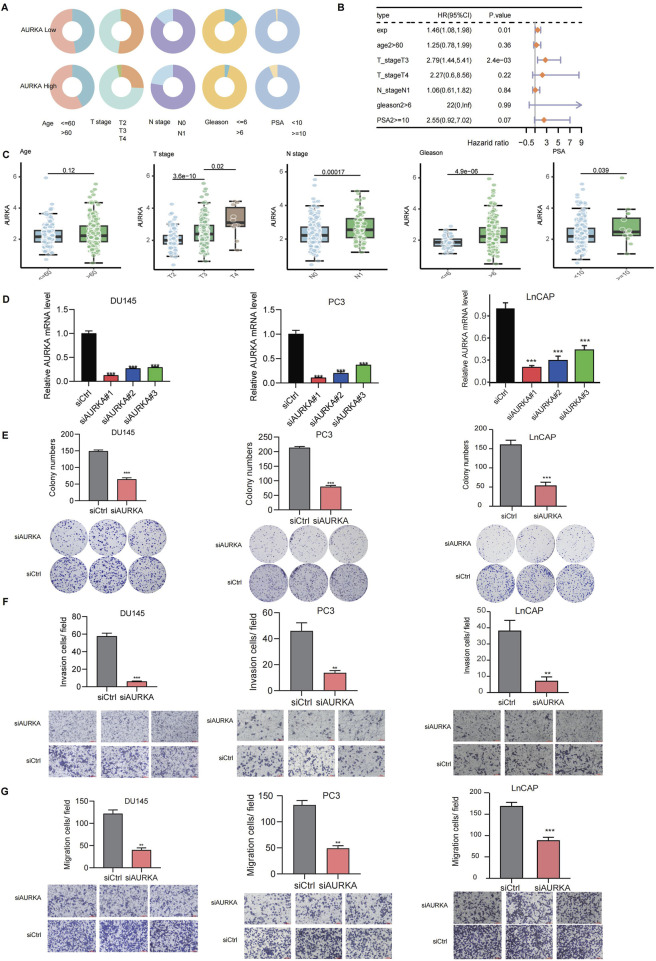
The clinical relevance and functional impact of the AURKA. **(A)** Donut chart of the association between AURKA and five clinicopathological variables (age, T-stage, N-stage, Gleason score, and PSA level). **(B)** Forest plot of multivariate survival analysis of AURKA in clinicopathological variables. **(C)** Histogram of the association between AURKA and five clinicopathological variables. **(D)** Relative AURKA mRNA level across siCtrl and siAURKA. **(E)** Colony formation assay showing inhibition upon AURKA knockdown in DU145, PC3, and LnCAP cell lines. Transwell assay demonstrating reduced invasion **(F)** and migration **(G)** after AURKA knockdown.

Based on the heterogeneity of AURKA expression in specific epithelial cell subsets, we investigated the relationship between AURKA expression and clinical information in PRAD. We found that the higher AURKA expression tended to be associated with higher T/N stage, Gleason score, and prostate-specific antigen (PSA) level (Figure 5A). The Gleason score is an important pathological indicator to evaluate the degree of differentiation and prognosis of PRAD. The higher the Gleason score, the more aggressive and malignant the tumor (Haddadin and Sun, 2025). The increased serum PSA level is one of the most important screening and diagnostic biomarkers for PRAD, and its level is associated with the tumor burden and progression of PRAD (Qiu et al., 2021). Further multivariate survival analysis showed that AURKA expression could be an independent risk factor for PRAD patients (HR = 1.46, 95% CI: 1.08–1.98, *p* = 0.01), indicating that high expression of AURKA was independently associated with a poorer survival prognosis (Figure 5B). With increasing T stage, the expression level of AURKA increased significantly (*p* = 3.6 × 10^−10^). Patients with a higher N stage (N1) had significantly higher AURKA expression than patients with a lower N stage (*p* = 0.00017). A higher Gleason score or PSA level was associated with significantly higher AURKA expression (Figure 5C).

To further validate the promoting influence of AURKA on the malignant phenotypes of PRAD cells, we performed *in vitro* experiments to assess the proliferation, migration, and invasion abilities of DU145, PC3, and LnCAP cell lines. In the proliferation experiment, the knockdown of AURKA significantly suppressed the proliferation ability of DU145, PC3, and LnCAP cells (Figure 5D). The colony formation experiment showed that the number of colonies in the siAURKA group was significantly lower than that in the control group (siCtrl) (Figure 5E). The invasion and migration experiment showed that the invasion and migration ability of DU145, PC3, and LnCAP cells were significantly decreased after the AURKA knockdown (Figures 5F,G).

Taken together, the results obtained from cell models are consistent with our conclusion of the tumor-promoting role of AURKA in prostate cancer development.

### 3.5 AURKA-based subpopulations population stratification aids prognosis prediction and combination therapy

**FIGURE 6:**
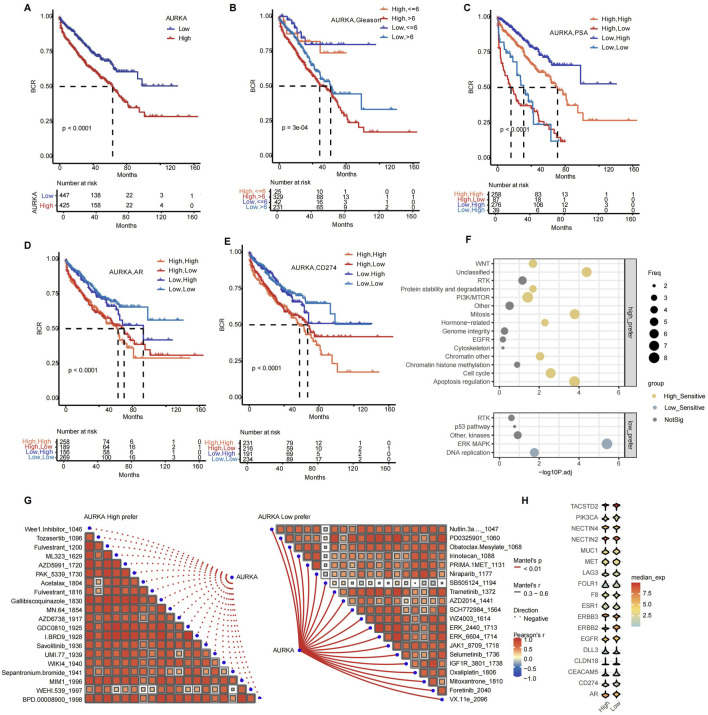
Correlation analysis of AURKA expression with prognosis, clinical characteristics, and drug sensitivity. Kaplan-Meier survival curve of subsets signatures, **(A)** AURKA low and high, **(B)** AURKA with Gleason score, **(C)** AURKA with PSA level, **(D)** AURKA with AR, **(E)** AURKA with CD274, with the optimal threshold for stratification. **(F)** Bubble plot of delta IC50 values between AURKA expression groups for drug target pathways. **(G)** Spearman correlation analysis between AURKA high pefer (left) and AURKA low pefer (right) scores. **(H)** Violin plot of the expression of ADC drug target genes across AURKA expression groups.

AURKA expression in combination with other indicators helps to stratify recurrence risk in patients with prostate cancer. By analyzing different datasets from TCGA as well as combined GEO data, we found that AURKA-high patients had a faster biochemical relapse (BCR) (Supplementary Figure 1; Figure 6A) and the AURKA-high cluster has a higher percentage of tumor-associated macrophage and Treg cell in TIME (Supplementary Figure 4A). In the combined survival analysis, the combination of AURKA expression and clinical factors can predict the prognosis of patients with more precision. For instance, the combined AURKA and Gleason score analysis showed that patients with high AURKA expression and high Gleason score had a worse prognosis. In contrast, lower Gleason score patients had a better BCR (Figure 6B). In addition, AURKA-low and PSA-low patients had longer survival, whereas AURKA+/PSA- or AURKA-/PSA + had a worse prognosis (Figure 6C). This finding suggested that AURKA may be a compensatory pathway for AR to support tumor activity. We conducted an analysis of the risk stratification of AURKA expression in combination with the common clinical targets AR and CD274 (PD-L1) expression (Figures 6D,E). The findings indicated that the most unfavorable prognosis was observed when both AURKA and the target exhibited high levels of expression. However, patients with AR-high or CD274-high with AURKA-low had a good prognosis.

Next, we analyzed the combination treatments that might benefit from AURKA inhibitors. We performed drug sensitivity analysis of different AURKA subsets. The IC50 values of the AURKA high-expression group for multiple drug-related pathways are significantly higher than those of the low-expression group, including WNT, Mitosis, Apoptosis regulation, and others. These pathways are associated with crucial biological processes, including cell cycle regulation, signal transduction, genomic stability, and apoptosis regulation (Figure 6F). This result correlated with different expression genes across differential AURKA levels (Supplementary Figures 4B,C). Patients with high expression of AURKA may be better suited for AURKA inhibitors combined with targeted therapy, such as kinase inhibitors (Wee1 inhibitors, savolitinib, AZD6738), apoptosis-targeting inhibitors (AZD5991, WEHI.539), and epigenetic & signaling pathway modulators (BRD9, ML323, WIKI4) (Figure 6G). At the same time, we evaluated the possibility of combination therapy of AURKA inhibitors with ADCs. The results revealed that TACSTD2, MET, ESR1, and ERBB3 were highly expressed in the AURKA high-expression group (Figure 6H).

In summary, AURKA-based population stratification contributed to personalized precision therapy, especially AURKA inhibitors with specific targeted therapy and antibody-drug conjugate (ADC) therapy may have better clinical benefit.

### 3.6 AURKA subgroup of genomic alteration differences and contributions for combination therapy strategies

**FIGURE 7:**
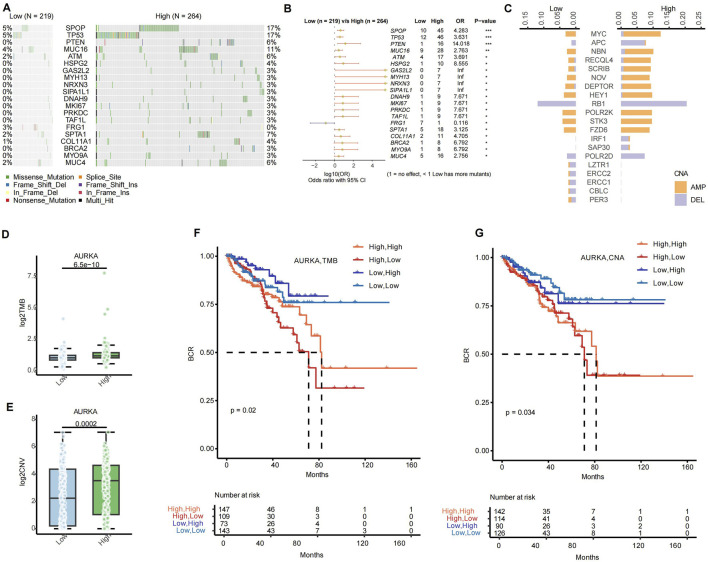
The disparities in DNA between the high and low expression groups of AURKA in the bulk RNA-seq. **(A)** Waterfall map presents of the AURKA high and low group. **(B)** Forest plot shows the gene mutation frequencies of the AURKA high and the low group. **(C)** Bar plot shows the CNA of the AURKA high and the low group, orange indicates amplification (AMP), and purple indicates deletion (DEL). Histogram of the association between AURKA and **(D)** TMB and **(E)** CNV. Kaplan-Meier survival curve of subsets signatures, **(F)** AURKA with CNA, **(G)** AURKA with TMB, with the optimal threshold for stratification. TMB, tumor mutation burden. CNV, copy number variation.

We conducted an analysis of DNA landscapes via AURKA high or low groups in PRAD. The subsequent genome-wide mutational analysis of PRAD revealed somatic mutations that differed between the high expression group (*n* = 264) and the low expression group (*n* = 219) of AURKA (Figure 7A). The mutation rate of the SPOP gene reached 17% in the high expression group, while it was only 5% in the low expression group. Furthermore, there were notable differences in the mutation frequencies of genes such as TP53 and PTEN between the two groups, which might reflect distinct tumor evolutionary trajectories. The mutation rate of the SPOP gene in the high expression group was found to be significantly higher than that in the low expression group (ratio = 4.283, *p* = 0.001). Additionally, the mutation rate of the TP53 gene in the high expression group was found to be significantly higher than that in the low expression group (ratio = 3.631, *p* = 0.001) (Figure 7B). In terms of copy number variations (CNAs), the high expression group of AURKA exhibited higher frequencies of gene amplification and deletion (Figure 7C).

Tumor mutational burden (TMB) is a pivotal factor in tumorigenesis and progression and serves as an essential biomarker for immunotherapy (Sundaresan et al., 2025). AURKA expression levels exhibited a substantial correlation with TMB, with the TMB in the high expression group demonstrating a significant increase compared to the low expression group (*p* = 6.5 × 10^−10^) (Figure 7D). Furthermore, AURKA-high/TMB-high patients had a relatively better prognosis than AURKA-high/TMB-low patients (Figure 7F). This finding indicated that AURKA-high patients may benefit from the combination of AURKA inhibitor and immunotherapy (Figures 3C, 6E). A high CNV burden has been associated with homologous recombination deficiency (HRD), which may affect tumor invasiveness, metastasis capacity, and response to PARPi treatment (Figure 7E) (Strickler et al., 2021). As demonstrated in Figure 7G, patients exhibiting high AURKA expression and a high CNV burden and a poorer prognosis (p = 0.034).

## 4 Discussion

AURKA has been validated as a driver of chromosomal instability and drug resistance in various tumors. In contrast, its role in the tumor microenvironment has not been fully understood in prostate cancer. In this study, we confirmed that AURKA overexpression is associated with an invasive tumor phenotype and poor prognosis in prostate cancer, consistent with pan-cancer (Shi et al., 2023). However, prostate cancer exhibits a unique dependence on AR and tumor stemness, which may enhance the role of AURKA in promoting genomic instability and maintaining tumor invasiveness after AR deprivation (Miralaei et al., 2021). AURKA-driven E2F etc. Pathways are conserved across tumors, but its interaction with the AR pathway in prostate cancer provides a complexity not seen in other malignancies (He et al., 2013). Consequently, further exploration of the role of AURKA in prostate cancer is required to guide precision therapy with AURKA inhibitors.

Our study revealed a heterogeneity of AURKA expression in prostate cancer that was previously not identified. The AURKA-high expressing subset mainly showed mitogenic activity and cell proliferation. However, the AURKA-low subgroup revealed an increased correlation between the AR and TNF-α pathways. This finding suggests that maintaining cells survival under the stress of treatment is an adaptive mechanism. Notably, AURKA overexpress patients with high PSA or low Gleason scores have a better prognosis. This complexity reflects a further interaction of AURKA subset cell with the tumor microenvironment that is worthy of further investigation. These findings contribute to a more nuanced understanding of the heterogeneity of AURKA subsets and the importance of molecular stratification.

AURKA inhibitors have limited efficacy as monotherapy in prostate cancer, consistent with observations in other solid tumors (Beltran et al., 2019). The efficacy of treatment can be limited owing to compensatory pathways such as AR reactivation and upregulation of DNA repair (Wu et al., 2025; Formaggio et al., 2021). The potential exists for combination strategies to capitalize on the role of AURKA in upregulating surface antigens or modulating immune evasion. High expression subset of AURKA may benefit from combination therapy with kinase inhibitors, or ICIs. TMB, CD274 expression was the biomarker of AURKA-high expression patients with PRAD for clinical outcome. Therefore, molecular stratification of AURKA subtypes may help AURKA inhibitors in clinical applications.

However, our study still has limitations. Our analysis was conducted exclusively using bulk data, which may lack an understanding of the longitudinal and spatial heterogeneity of AURKA. In addition, *in vivo* and *in vitro* experiments, and clinical cohort validation with specific treatments are needed.

## 5 Conclusion

In summary, our study highlighted the various oncogenic roles of AURKA in prostate cancer, and the heterogeneity of its expression has different molecular characteristics and clinical prognosis. AURKA expression-based subset for precise stratification can facilitate personalized treatment. In the future, clinical precision therapy will entail the combination of various combination therapy strategies based on different stratifications.

## Data Availability

The original contributions presented in the study are included in the article/Supplementary Material, further inquiries can be directed to the corresponding authors.

## Abbreviations

AURKA, aurora kinase-A; PRAD, prostate adenocarcinoma; TIME, tumor immune microenvironment; CSC, cancer stem cell; TMB, tumor mutational burden; HRD, homologous recombination deficiency; PARPi, PARP inhibitor; ICB, immune checkpoint blockade; AR, androgen receptor; TNF-α, tumor necrosis factor-α; EMT, epithelial-mesenchymal transition; ADC, antibody-drug conjugate; GDSC, genomics of drug sensitivity in cancer; scRNA-seq, single-cell RNA sequencing; GI, gastrointestinal; TGCT, testicular germ cell tumor; LUAD, lung adenocarcinoma; BRCA, breast cancer; THCA, thyroid cancer; KIRP, kidney renal papillary cell carcinoma; CHOL, cholangiocarcinoma; SNV, single-nucleotide variant; COAD, colon adenocarcinoma; ACC, adrenocortical carcinoma; READ, rectum adenocarcinoma; OV, ovarian cancer; KEGG, Kyoto encyclopedia of genes and genomes; GEO, gene expression omnibus; CNAs, copy number variations; SVs, structural variants.
