# Supplementary figures and images for: Aurora kinase-a expression heterogeneity and potential benefit of combination therapy in prostate adenocarcinoma
Source: Front Cell Dev Biol. 2025 Jul 11;13:1608711. doi: 10.3389/fcell.2025.1608711 (PMC12289701; doi:10.3389/fcell.2025.1608711)

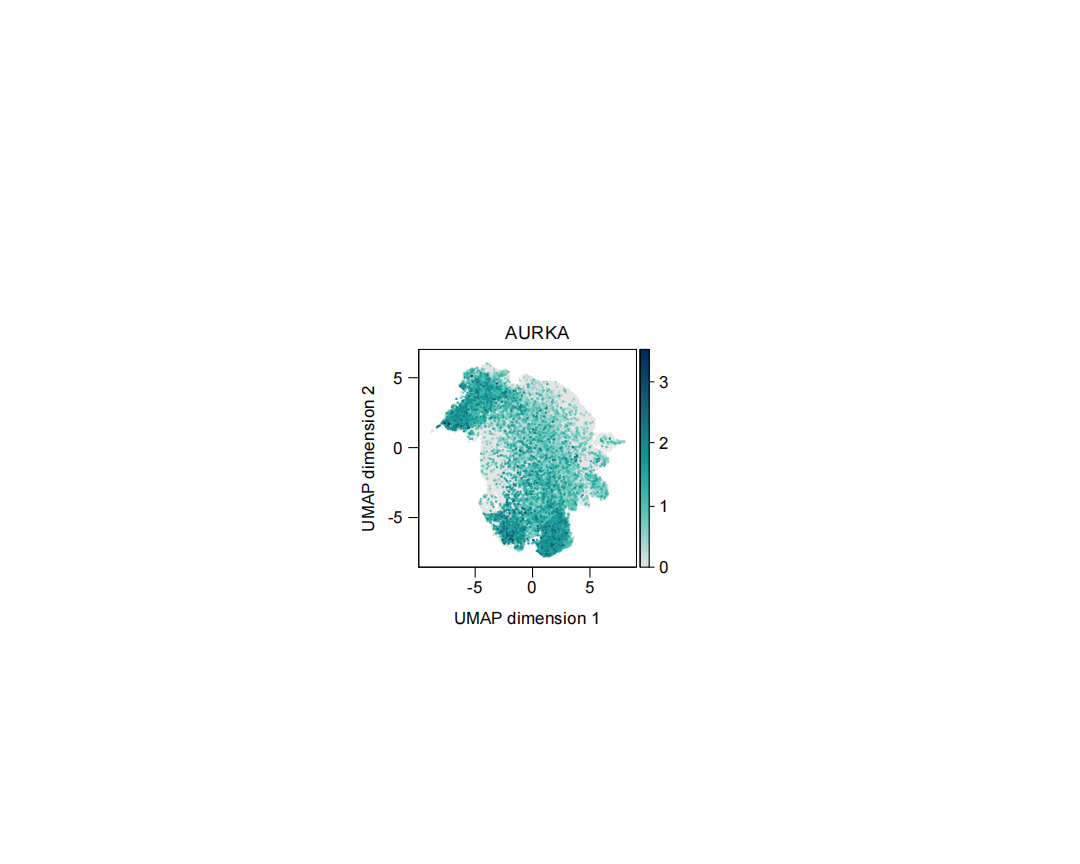

Supplement: Supplementary file 1 [file Image3.tif]

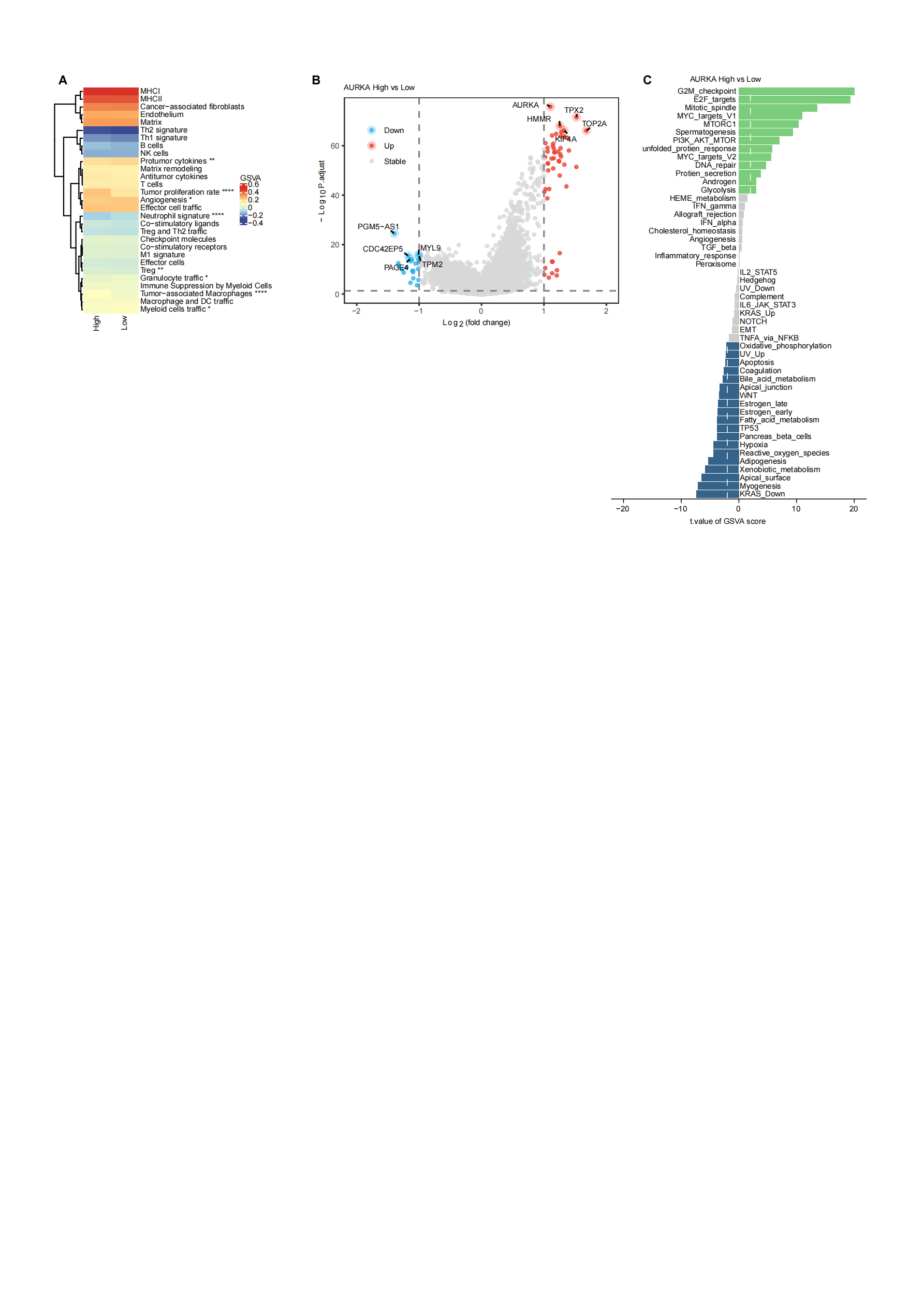

Supplement: Supplementary file 2 [file Image4.tif]

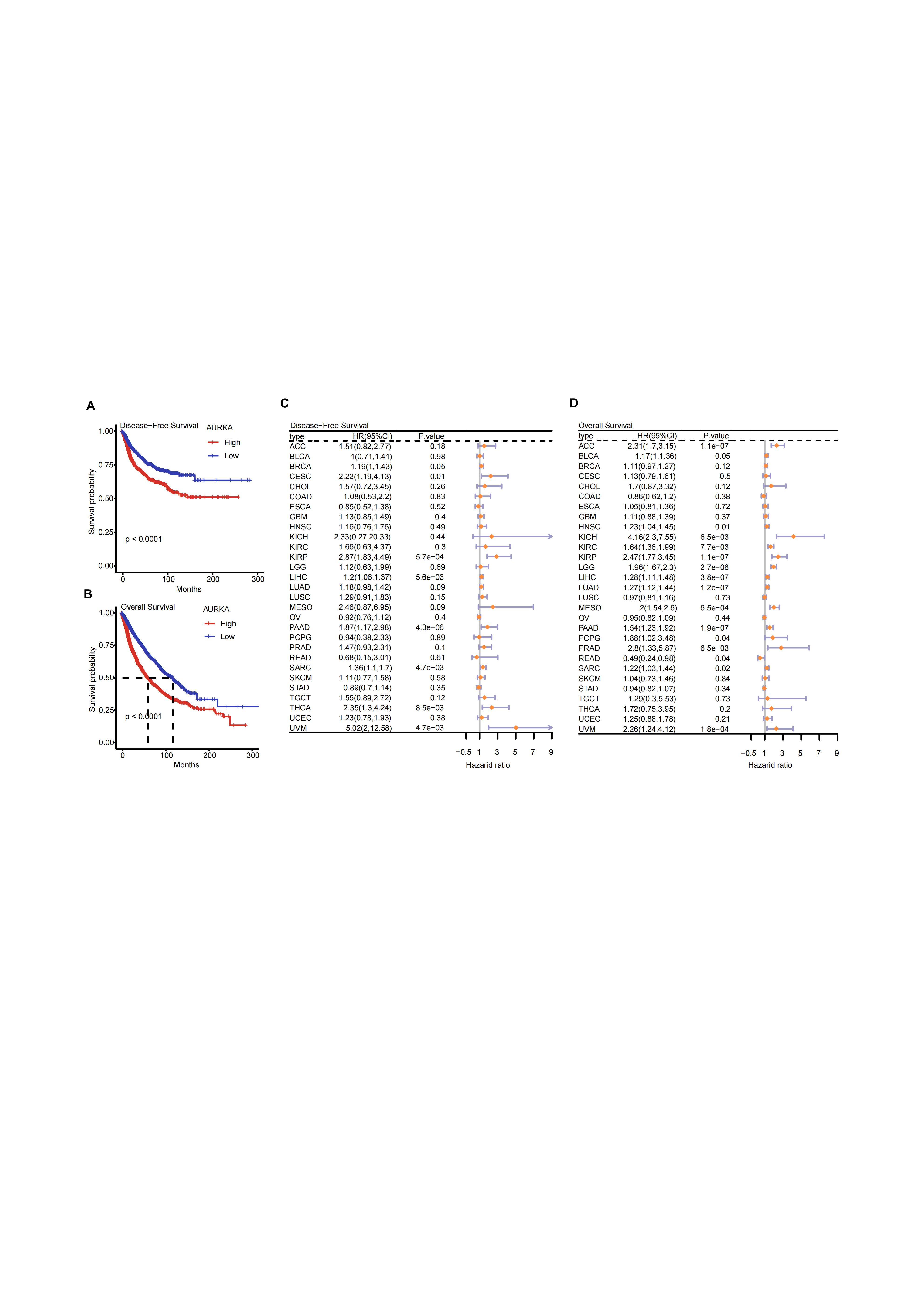

Supplement: Supplementary file 3 [file Image1.jpeg]

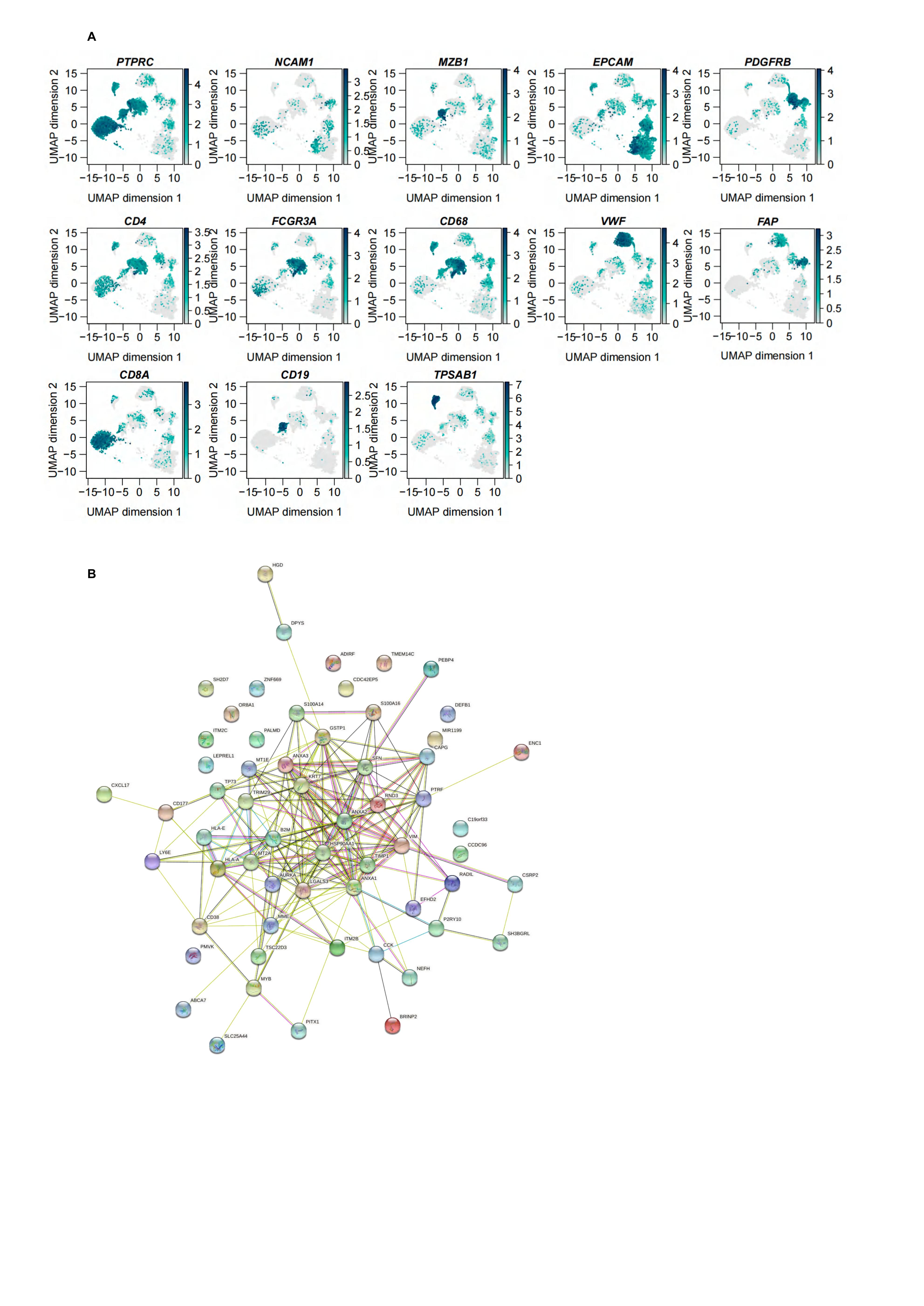

Supplement: Supplementary file 4 [file Image2.jpeg]
